# A rare manifestation of burns after lightning strike in rural Ghana: a case report

**DOI:** 10.1186/s13256-017-1378-0

**Published:** 2017-07-25

**Authors:** Paschal Awingura Apanga, John Atigiba Azumah, Joseph Bayewala Yiranbon

**Affiliations:** Talensi District Hospital, Ghana Health Service, Upper East Region, Tongo, Ghana

**Keywords:** Burns, Lightning, Rare, Vaseline, Silver sulfadiazine

## Abstract

**Background:**

Lightning is a natural phenomenon that mostly affects countries in the tropical and subtropical regions of the globe, including Ghana. Lightning strikes pose a global public health issue. Although strikes to humans are uncommon, it is associated with high morbidity and mortality.

**Case presentation:**

We present a case of a 10-year-old Ghanaian girl who got second-degree burns after being struck by lightning. She was put on an intravenous broad-spectrum antibiotic (ceftriaxone), Ringer’s lactate, and her burns were dressed with sterile gauze impregnated with Vaseline (petroleum jelly) and silver sulfadiazine ointment. There was marked improvement on the 16^th^ day of treatment despite the lack in capacity of the hospital to carry out some laboratory diagnostic tests. On the 21^st^ day of treatment, the burns were completely healed without scars and contractures.

**Conclusions:**

This is evidence of burns due to lightning strike, despite its rare occurrence. This report will help inform those in doubt, particularly in communities where lightning injuries are associated with widespread superstition. The case report also revealed how rural healthcare can be challenging amid a lack of basic diagnostic equipment and logistics. However, in resource-limited settings, Vaseline (petroleum jelly) and silver sulfadiazine could be used in the treatment of burns.

## Background

Lightning is discharge of electrical current as a result of an imbalance between the electrical charge of the clouds and the Earth’s surface [1]. It is a powerful and spectacular natural phenomenon, and usually occurs when there is a difference in voltage of 30,000 V or higher, which exceeds the inherent resistance [1, 2]. Usually thunder, a sound produced by lightning, follows it after it strikes the Earth’s surface. Lightning is a global public health issue and is the second leading cause of weather-related deaths [3, 4]. It accounts for about 24,000 deaths and 240,000 injuries annually [5].

Lightning mostly occurs during summer and, in countries like Ghana, it occurs during the rainy season [6, 7]. It strikes the Earth’s surface more than eight million times a day [1]. Lightning-related injuries are more common in the young, with a vulnerable age group of 10–29 years [8]. Males have a five times greater risk of being struck by lightning than their female counterparts [9]. Lightning usually occurs during outdoor activities rather than indoors. Individuals engaging in outdoor activities such as fishing golfing, camping, swimming, boating, hiking, and so on, are more prone to strikes by lightning [1, 10]. However, indoor strikes by lightning have also been reported [9]. The risk of being struck by lightning is dependent, therefore, on seasonal, regional, and temporal factors [3].

Lightning injuries are uncommon in humans, although long-term morbidity and high mortality has been reported [7]. Lightning strikes can cause ear damage, brain damage, cardiac failure, blunt trauma, neurological syndromes, muscle injuries, eye injuries, skin lesions, and burns [1]. Unlike other high-voltage-related accidents, lightning injuries do not only involve high-voltage electricity, but they can also occur with a very short exposure time to the electric current. Hence, lightning injuries are not only as a result of the electric current, but may be due to high temperatures and blast waves [11, 12]. Lightning injuries can affect the human body through various mechanisms. It may affect the body by direct strike, side splash, contact, blunt trauma, ground current, and injury by a weak upward streamer [13]. We report the first case of a patient with burns from lightning strike in rural Ghana who reported to a hospital for treatment.

## Case presentation

**Fig. 1:**
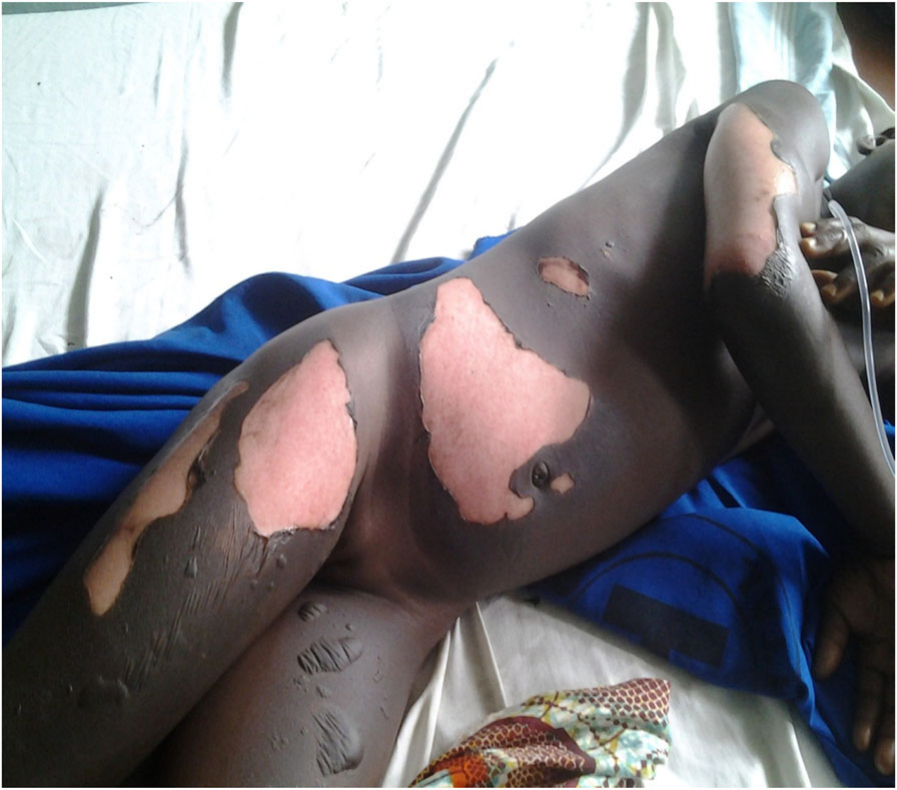
Image showing burns from lightning strike on the first day of presentation

A 10-year-old Ghanaian girl presented to a hospital with burns on her right arm, abdomen and both thighs after she was struck by lightning during a downpour. She was struck by lightning when she opened her bedroom door to walk out. On general examination, she weighed 25 kg and was febrile with a temperature of 38.0 °C, not pale, not dehydrated, and not jaundiced. Her blood pressure (100/70 mmHg), pulse (80 beats per minute) and respiratory rates (16 cycles per minute) were normal. She sustained second-degree burns on the posterior part of her right arm, abdomen, anterolateral part of her right thigh and the anteromedial part of her left thigh as shown in Fig. 1. Using the “rule of nines” for estimating the total body surface area for burns of persons at least 10 years old [14], the estimated burnt surface area was 9%. The affected areas of the burns were erythematous with blisters. However, examinations of her gastrointestinal, cardiovascular, and neurological systems were all normal.

Our patient was admitted to the children’s ward and initial laboratory results showed leukocytosis of 15 × 10^9/^L with neutrophilia of 80%. Serum urea (4 mmol/L), creatinine (80 μmol/L), sodium (138 mmol/L), and potassium (4.1 mmol/L) levels were normal. A routine urine test was normal. However, the hospital lacked the capacity to carry out wound swabs and blood tests for culture and sensitivity. She was put on an intravenous broad-spectrum antibiotic (ceftriaxone 1 g daily) for 7 days and oral paracetamol 500 mg every 8 hours for 5 days. She was also given 25 mg diclofenac every 8 hours for 5 days to relieve pain. A urethral catheter was inserted to monitor her urine output while she was given 900 mL of Ringer’s lactate intravenously for the first 24 hours to ensure that she remained hemodynamically stable. Half of the volume of fluid (450 mL) was administered in the first 8 hours and the remainder was given in the next 16 hours. The burns were dressed daily with sterile gauze impregnated with Vaseline (petroleum jelly) and silver sulfadiazine. In addition, our patient was covered with a blanket to prevent hypothermia. Our patient had physiotherapy daily to prevent contractures of her affected limbs.

**Fig. 2:**
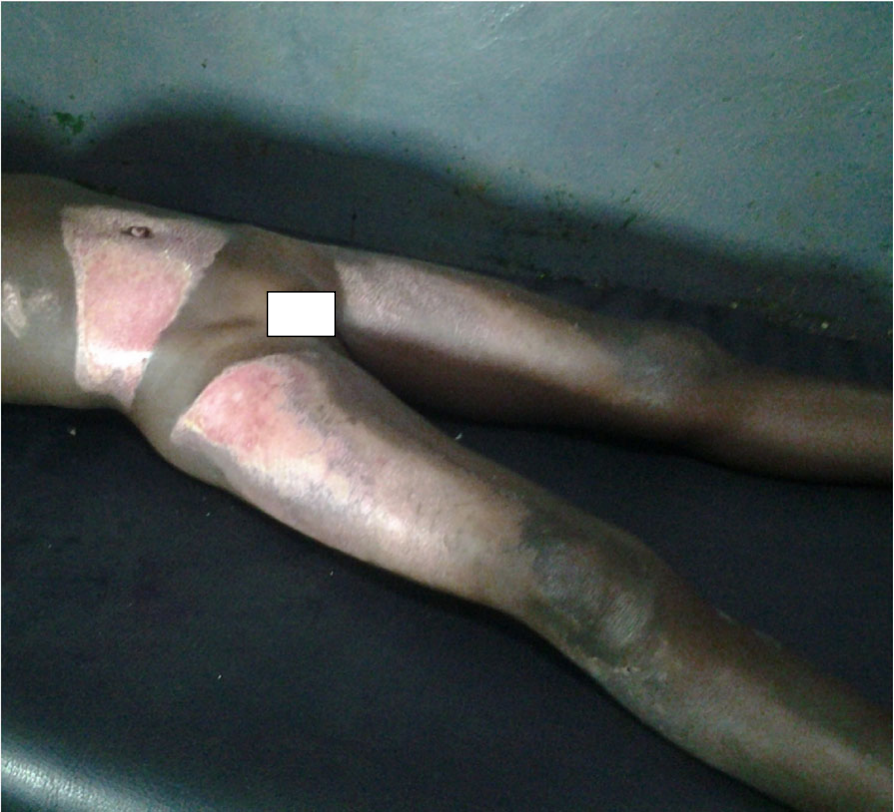
Image on the 16^th^ day of treatment showing the healing of burns

After 48 hours of treatment, her temperature became normal. Treatment was continued and her vital signs were checked regularly and remained normal. On the 16^th^ day of treatment in the ward, the burns had almost completely healed and there were no contractures as shown in Fig. 2. The full blood count was repeated and it was found to be normal. Our patient was discharged from the ward and put on oral amoxicillin 500 mg every 8 hours for 5 days. She and her guardian were asked to return to the hospital daily for dressing as their residence was close to the hospital. On the 21^st^ day of treatment, the burns were completely healed and treatment was discontinued.

## Discussion

This case report is unusual as the incidence of burns as a result of lightning strike is rare in Ghana. Most cases of burns reported are mainly due to fires, chemicals, electricity, and hot liquids [15, 16]. In many sub-Saharan African countries, including Ghana, lightning injuries have largely been blamed on various misconceptions including religious beliefs and superstitions, and afflicted individuals may face social stigma [4, 17, 18]. However, this is misleading as lightning injuries have been well documented scientifically [1].

The lightning injury in this study occurred when our patient tried to step out of her bedroom. This finding is contrary to many studies that reported lightning strikes during outdoor activities [1, 10, 19, 20]. However, our finding was consistent with studies by Mistovich *et al*., who reported lightning strikes that occurred indoors [9]. Opening of doors when indoors has been found to be a risk factor for lightning injuries [9], this might have accounted for the lightning strike in this study.

The fever, high white blood cell count, and leukocytosis from the initial laboratory results were suggestive of a bacterial infection. However, the hospital lacked the capacity to carry out wound swabs and blood tests for culture and sensitivity, which might have informed clinicians of the focus of infection and the type of bacterial organism involved. The lack of diagnostic logistics and equipment reemphasizes the challenges clinicians encounter in providing primary healthcare in poorly resourced settings [21]. In spite of the challenges encountered by the clinicians, the parenteral administration of ceftriaxone as well as the gauze dressing impregnated with Vaseline and silver sulfadiazine seems to have brought the fever, high white blood cell, and leukocytosis under control to normal levels. The use of sterile gauze dressing impregnated with Vaseline and silver sulfadiazine was successful in treating the burns. Similar studies have found silver sulfadiazine to be effective in the treatment of burns [22]. On the contrary, some studies have reported hypersensitivity reactions, wound contracture, and scar formation associated with the usage of silver sulfadiazine in the treatment of burns [14, 23]. This was not found in our study and might probably be due to the addition of Vaseline to silver sulfadiazine during the gauze dressings.

## Conclusions

This report has provided evidence of a case of a patient with burns resulting from lightning strike, despite its rare occurrence. This also serves as evidence for those in doubt, particularly in communities where lightning injuries are associated with widespread superstition. The case report also revealed how challenging providing primary care in poorly resourced settings can be amid lack of basic diagnostic equipment and logistics. However, we recommend that in resource-limited settings, Vaseline (petroleum jelly) and silver sulfadiazine can be used in the treatment of burns.
